# Guideline-indicated treatments and diagnostics, GRACE risk score, and survival for non-ST elevation myocardial infarction

**DOI:** 10.1093/eurheartj/ehy517

**Published:** 2018-09-07

**Authors:** Marlous Hall, Owen J Bebb, Tatandashe B Dondo, Andrew T Yan, Shaun G Goodman, Hector Bueno, Derek P Chew, David Brieger, Philip D Batin, Michel E Farkouh, Harry Hemingway, Adam Timmis, Keith A A Fox, Chris P Gale

**Affiliations:** 1 Leeds Institute of Cardiovascular and Metabolic Medicine, University of Leeds, Worsley Building, Level 11, Clarendon Way, Leeds, UK; 2 Cardiology Department, York Teaching Hospital NHS Foundation Trust, Wigginton Road, York, UK; 3 Department of Medicine, Terrence Donnelly Heart Centre, St. Michael's Hospital, University of Toronto, 30 Bond Street, Toronto, Ontario, Canada; 4 Centro Nacional de Investigaciones Cardiovasculares (CNIC), Calle de Melchor Fernandez Almagro, 3, s/n, Madrid, Spain; 5 Instituto de investigación i+12 and Cardiology Department, Hospital Universitario 12 de Octubre, s/n, Madrid, Spain; 6 Facultad de Medicina, Universidad Complutense de Madrid, Plaza de Ramon y Cajal, s/n, Madrid, Spain; 7 Cardiology Department, Flinders Medical Centre and Flinders University, Flinders Drive, Bedford Park, Adelaide, SA, Australia; 8 Cardiology Department, Concord Repatriation General Hospital, Hospital Road, Concord, Sydney, NSW, Australia; 9 Cardiology Department, The Mid Yorkshire Hospitals NHS Trust, Aberford Road, Wakefield, UK; 10 Peter Munk Cardiac Centre and Heart and Stroke Richard Lewar Centre, University of Toronto, David Naylor Building, 6 Queen's Park Cres W, Toronto, Ontario, Canada; 11 Research Department of Clinical Epidemiology, The Farr Institute of Health Informatics Research, University College London, 222 Euston Road, Kings Cross, London, UK; 12 The National Institute for Health Research, Biomedical Research Centre, University College London Hospitals NHS Foundation Trust, University College London, 170 Tottenham Court Road, London, UK; 13 Cardiology Department, Barts Health Centre, Queen Mary University, W Smithfield, London, UK; 14 Centre for Cardiovascular Science, University of Edinburgh, Old College South Bridge, Edinburgh, UK

**Keywords:** Non-ST-elevation myocardial infarction, Quality of care, Mortality, GRACE risk score

## Abstract

**Aims:**

To investigate whether improved survival from non-ST-elevation myocardial infarction (NSTEMI), according to GRACE risk score, was associated with guideline-indicated treatments and diagnostics, and persisted after hospital discharge.

**Methods and results:**

National cohort study (*n* = 389 507 patients, *n* = 232 hospitals, MINAP registry), 2003–2013. The primary outcome was adjusted all-cause survival estimated using flexible parametric survival modelling with time-varying covariates. Optimal care was defined as the receipt of all eligible treatments and was inversely related to risk status (defined by the GRACE risk score): 25.6% in low, 18.6% in intermediate, and 11.5% in high-risk NSTEMI. At 30 days, the use of optimal care was associated with improved survival among high [adjusted hazard ratio (aHR) −0.66 95% confidence interval (CI) 0.53–0.86, difference in absolute mortality rate (AMR) per 100 patients (AMR/100–0.19 95% CI −0.29 to −0.08)], and intermediate (aHR = 0.74, 95% CI 0.62–0.92; AMR/100 = −0.15, 95% CI −0.23 to −0.08) risk NSTEMI. At the end of follow-up (8.4 years, median 2.3 years), the significant association between the use of all eligible guideline-indicated treatments and improved survival remained only for high-risk NSTEMI (aHR = 0.66, 95% CI 0.50–0.96; AMR/100 = −0.03, 95% CI −0.06 to −0.01). For low-risk NSTEMI, there was no association between the use of optimal care and improved survival at 30 days (aHR = 0.92, 95% CI 0.69–1.38) and at 8.4 years (aHR = 0.71, 95% CI 0.39–3.74).

**Conclusion:**

Optimal use of guideline-indicated care for NSTEMI was associated with greater survival gains with increasing GRACE risk, but its use decreased with increasing GRACE risk.

## Introduction

For patients with non-ST-elevation myocardial infarction (NSTEMI), evidence from international studies suggests that guideline-indicated care treatment and diagnostics are associated with improved clinical outcomes.^1–4^ Evidence from randomized controlled trials, suggest that the absolute effect is greater for NSTEMI at high ischaemic risk where there is reduced mortality, and lower rates of unscheduled revascularization, stroke, and hospitalization for heart failure.^5–8^ It is unknown, however, if beyond the setting of trials the effects of such interventions for NSTEMI (including pharmacotherapies as well as an invasive coronary strategy) are evident and, if so, whether such effects persist after discharge from hospital.

The Myocardial Ischaemia National Audit Project (MINAP) represents all acute hospitals in the single healthcare system of England and Wales and prospectively collects information about treatments provided, case mix and mortality of patients hospitalized with acute coronary syndrome over 15 years.^9^^,^^10^ Thus, MINAP is an optimal research conduit for understanding the impact of evidence-based NSTEMI care on clinical outcomes. We accessed anonymized patient data from MINAP to investigate whether improved survival associated with the use of NSTEMI guideline-indicated treatments was evident across the spectrum of NSTEMI risk, and whether mortality benefits were maintained over the long-term following discharge from hospital.

## Methods

### Data and subjects

The study was conducted using the MINAP. Data were entered electronically at each hospital where they were encrypted prior to secure transfer to a central database, anonymized and then distributed upon application for research. Each year MINAP data are validated at participating hospitals.^9^

**Figure 1:**
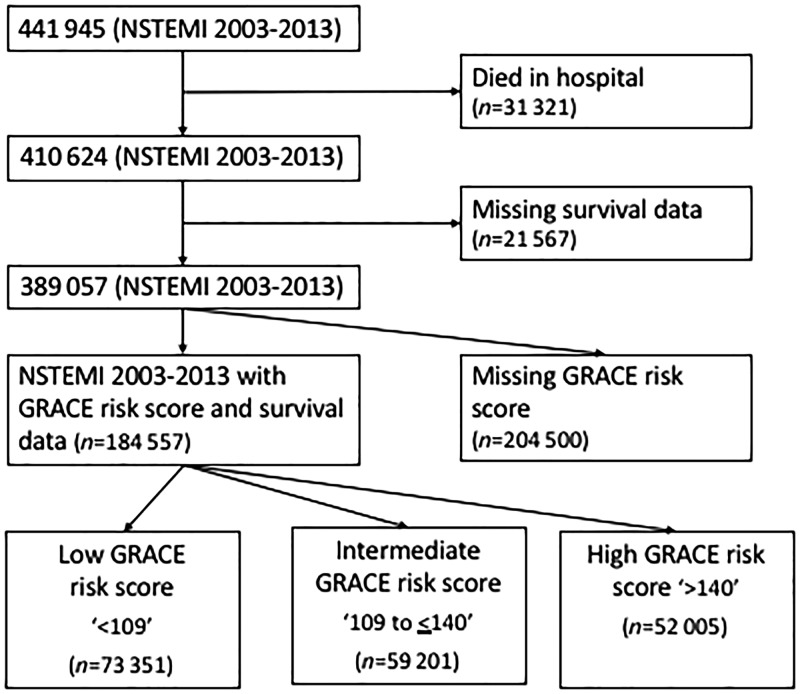
Consort diagram of exclusions of the Myocardial Ischaemia National Audit Project (MINAP) dataset.

The study population included all patients (1 January 2003 to 30 June 2013) with a discharge diagnosis of NSTEMI. This diagnosis was determined by the treating team and based upon clinical presentation and investigations, including biomarkers, in keeping with the universal definition of myocardial infarction.^11^ Patients who died in hospital (*n* = 31 321) and for whom, there were no survival data (*n* = 21 567) were excluded from the cohort in keeping with previous work (*Figure 1*).^3^

Baseline clinical risk was determined according to the adjusted mini-GRACE risk score, which has been validated using MINAP data and endorsed by the National Institute for Health and Care Excellence (NICE).^12^^,^^13^ The variables included age, cardiac arrest, electrocardiographic ST-segment deviation, elevated cardiac enzymes, systolic blood pressure and heart rate at the time of hospitalization, use of a loop diuretic (substituted for Killip Class), and creatinine. In line with the American Heart Association/American College of Cardiology and European Society of Cardiology NSTEACS guidelines,^14^^,^^15^ we categorized patients according to their risk of in-hospital mortality using the calculated GRACE risk score as low (<109; predicted mortality <1.0%), intermediate (≥109 to ≤140; predicted mortality ≥1.0% to ≤3.0%), and high (>140; predicted mortality >3.0%).

Receipt of guideline indicated care was measured according to a composite optimal care variable. This comprised 13 care interventions, previously mapped to MINAP data by the authors,^2^ which were identified following review of international guidelines.^16–20^ The 13 interventions included receipt, if eligible, of an electrocardiogram pre- or in-hospital, pre-hospital receipt of aspirin, echocardiography, an aldosterone antagonist during admission, coronary angiography, aspirin on discharge, P2Y_12_ inhibition on discharge, ACE inhibitors (ACEi)/angiotensin receptor blockers (ARBs) on discharge, β-blocker on discharge, HMG Co-A reductase inhibitor (statin) on discharge, referral for cardiac rehabilitation, smoking cessation advice, and dietary advice (see Supplementary material online, *Section S1*).^2^^,^^3^ Patients were classified as ineligible if a treatment was listed as contraindicated, not indicated, not applicable, if the patient declined treatment as recorded in MINAP or if the patient was hospitalized prior to the publication year of treatment recommendation in the guidelines. If patients were deemed eligible, but there was no data regarding receipt, they were assumed to have not received that intervention. Optimal care was defined at the individual patient level, if they received all of the care opportunities for which an individual patient was eligible; thus, patients missing one or more eligible care opportunities were assigned to the suboptimal care group. Patients who were listed as having a contraindication for one care intervention were not eligible for that care intervention and only the care interventions for which a patient was eligible for were considered in the calculation of the optimal care variable.

We also studied the impact of optimal care compared with suboptimal care for components of the care pathway separately, including pharmacological therapies [pre-hospital receipt of aspirin, aldosterone antagonist during admission, aspirin on discharge, P2Y_12_ inhibition on discharge, ACEi/ARBs on discharge, β-blocker on discharge and HMG Co-A reductase inhibitor (statin) on discharge), investigative and invasive coronary strategies (receipt of a pre- or in-hospital electrocardiogram, echocardiography and coronary angiography), and lifestyle care opportunities (referral for cardiac rehabilitation, receipt of smoking cessation advice and receipt of dietary advice)].

The primary outcome measure was all-cause mortality after discharge from hospital up to the maximum follow-up time of 8.4 years [median 2.3, interquartile range (IQR) 1.1–3.9 years], which represented over 1 079 044 person years. Mortality data were obtained via linkage to the United Kingdom national death records held by the Office for National Statistics.

### Statistical analysis

Baseline characteristics were described using numbers and percentages for categorical data and means and standard deviations or medians and IQRs for normal and non-normally distributed continuous variables. Differences in patient characteristics according to patient demographic and baseline clinical data were compared across GRACE risk score categories using *χ*^2^, *t*-tests, and Wilcoxon rank-sum tests as appropriate for the data type and distribution.

Flexible parametric survival modelling^21^ was used to assess the association of optimal care with long-term survival according to GRACE risk score category. To model the change in hazard ratio (HR) over continuous follow-up time, optimal care, and GRACE risk score categories were included in the model as time-varying covariates. We selected flexible parametric survival modelling to overcome violation of the proportional hazards assumption and to estimate the baseline hazard function using restricted cubic splines (see Supplementary material online, *Section S2*, *Table S2 for model selection choice*). The model was adjusted for patient demographics (sex, Index of Multiple Deprivation), medical history [diabetes, smoking status, family history of coronary heart disease, hypertension, previous myocardial infarction, previous angina, peripheral vascular disease, cerebrovascular disease, chronic obstructive pulmonary disease or asthma, chronic renal failure (defined as creatinine chronically >200 µmol/L (>2.26 mg/dL)), congestive cardiac failure, previous percutaneous coronary intervention, coronary artery bypass graft surgery, and total cholesterol]. Survival differences were quantified as HRs and differences in absolute mortality rates (AMR) per 100 patients, with an AMR of zero indicating no differences in mortality rates between the optimally treated patients (received all care interventions they were eligible for) vs. those sub-optimally treated. However, an AMR less than zero indicates lower mortality rates in the optimally managed patients compared with the sub-optimally managed.

Multiple imputation by chained equations was used to produce 10 imputed datasets to minimize potential bias caused by missing data. Of the 34 MINAP variables considered in the study (*Table 1*), the majority had less than 10% missing data (*n* = 26). Overall, we had complete data across all variables included in the flexible parametric modelling for 184 390 of patients. Multiple imputation by chained equations allowed the inclusion all 389 057 patients in the main study analyses and, as such, prevented loss of information whilst mitigating potential bias from missing data. Pooled model estimates and accompanying 95% confidence intervals (CIs) were generated according to Rubin’s rules (see Supplementary material online, *Section S3*, *Table S3*).^22^ All imputed data were compared with complete case analyses according to imputation good practice guidelines (see Supplementary material online, *Table S4*).^23^ All tests were two-sided, and statistical significance was considered as *P* < 0.05. Statistical analyses were performed in Stata MP64 version 14 (http://www.stata.com/) and R version 3.1.2 (https://cran.r-project.org/).

**Table 1:** Baseline characteristics and care interventions received for all NSTEMI and by GRACE risk score category

	Analytical cohort (*n* = 389 057)	GRACE risk score category (*n* = 184 557)^a^			*P*-value for difference between GRACE risk score category	Missing data (*n*, % of analytical cohort)
		Low (<109) (*n* = 73 351) (39.7%)	Intermediate (109 to <140) (*n* = 59 201) (32.1%)	High (>140) (*n* = 52 005) (28.2%)		
Patient demographics						
Age (years), median (IQR)	72.7 (61.7–81.2)	59.5 (52.0–66.0)	76.0 (70.4–81.0)	84.0 (79.0–88.0)	NA	638 (0.2)
Sex (males), *n*(%)	244 837 (63.1)	53 818 (73.4)	35 442 (59.9)	27 104 (52.1)	<0.001	258 (0.1)
Patient medical history and clinical measures						
History of ischaemic heart disease^b^, *n* (%)	162 064 (45.2)	22 885 (31.4)	29 334 (50.0)	28 676 (55.7)	<0.001	23 879 (6.1)
Hypertension, *n* (%)	188 503 (48.5)	33 872 (46.5)	94 894 (59.4)	30 605 (59.5)	<0.001	25 991 (6.7)
Diabetes mellitus, *n* (%)	81 469 (20.9)	13 229 (18.2)	15 771 (26.9)	13 598 (26.5)	<0.001	27 712 (7.1)
Dyslipidaemia, *n* (%)	121 243 (33.7)	27 292 (38.0)	21 893 (37.9)	15 952 (31.6)	<0.001	28 771 (7.4)
Family history of IHD, *n* (%)	77 288 (26.2)	29 184 (44.0)	12 302 (25.0)	5915 (14.8)	<0.001	94 215 (24.2)
Smoking status (current or previous smoker vs. never smoked), *n* (%)	217 116 (60.3)	49 323 (68.6)	33 327 (59.0)	25 589 (53.1)	<0.001	29 219 (7.5)
Peripheral vascular disease, *n* (%)	18 324 (5.2)	2181 (3.1)	3431 (6.0)	3301 (6.5)	<0.001	34 467 (8.9)
Chronic heart failure, *n* (%)	24 529 (6.9)	1205 (1.7)	3759 (6.4)	7683 (15.0)	<0.001	33 304 (8.6)
COPD or asthma, *n* (%)	56 708 (14.6)	9176 (12.8)	10 796 (18.6)	10 176 (20.1)	<0.001	33 633 (8.6)
Chronic kidney disease, *n* (%)	21 938 (6.2)	1637 (2.3)	4349 (7.4)	7216 (14.1)	<0.001	33 448 (8.6)
Cerebrovascular disease, *n* (%)	34 146 (9.6)	3505 (4.8)	7146 (12.2)	7867 (15.3)	<0.001	34 302 (8.8)
Heart rate (b.p.m.), median (IQR)	80 (67 -95)	74.0 (64.0–86.0)	79.0 (66.0–92.0)	89.0 (74.0–107.0)	NA	65 863 (16.9)
Systolic blood pressure (mmHg), mean (SD)	142.5 (28.4)	149.1 (26.5)	144.3 (27.3)	131.0 (27.0)	NA	66 688 (17.1)
Cardiac arrest (pre-hospital), *n* (%)	1305 (0.7%)	99 (0.1)	354 (0.6)	852 (1.6)	NA	22 901 (5.9)
Initial creatinine (µmol/L), median (IQR)	92.0 (76.0–114.0)	84.0 (72.0–98.0)	94.0 (78.0–117.0)	110.0 (86.0–144.0)	NA	165 622 (42.6)
ST-deviation on admission, *n* (%)	108 189 (30.62)	12 117 (16.5)	16 262 (27.5)	22 720 (43.7)	NA	35 699 (9.2)
Care interventions						
ECG during admission, *n* (%)	371 149 (95.4)	73 351 (100)	59 201 (100)	52 005 (100)	No difference	9295 (4.7)
Receipt of pre-hospital aspirin^c^, *n* (%)	91 679 (70.8)	21 681 (71.1)	13 915 (60.0)	8721 (47.1)	<0.001	4917 (3.7)
Echocardiogram^c^, *n* (%)	207 128 (53.3)	44 772 (61.0)	36 834 (62.2)	32 404 (62.3)	<0.001	11 053 (2.8)
Receipt of angiography^c^, *n* (%)	198 303 (55.7)	60 063 (85.4)	34 691 (65.7)	15 903 (38.0)	<0.001	15 656 (4.0)
Aspirin on discharge^c^, *n* (%)	301 639 (88.46)	56 130 (92.2)	45 626 (92.6)	39 458 (92.1)	0.95	32 983 (8.5)
P2Y_12_ inhibition on discharge^c^, *n* (%)	127 315 (93.1)	39 858 (95.7)	31 105 (93.1)	24 867 (89.3)	<0.001	4236 (1.1)
ACEi/ARB on discharge^c^, *n* (%)	169 942 (78.9)	15 400 (91.5)	21 360 (89.9)	26 715 (86.2)	<0.001	206 719 (53.1)
β-Blocker on discharge^c^, *n* (%)	138 656 (78.8)	7625 (92.3)	14 730 (90.5)	24 953 (88.4)	<0.001	5300 (1.4)
Receipt of aldosterone antagonist during admission^c^, *n* (%)	2004 (17.5)	191 (13.4)	614 (19.4)	990 (20.3)	<0.001	4798 (1.2)
Statin on discharge^c^, *n* (%)	297 045 (85.4)	55 965 (91.4)	45 909 (90.8)	38 916 (87.4)	<0.001	4519 (1.2)
Referral for cardiac rehabilitation^c^, *n* (%)	279 027 (76.0)	60 450 (86.1)	44 508 (81.5)	33 671 (74.6)	<0.001	4519 (1.2)
Smoking cessation advice received^c^, *n* (%)	32 109 (19.4)	17 405 (48.7)	5434 (29.47)	2350 (18.4)	<0.001	11 658 (3.0)
Dietary advice received^c^, *n* (%)	119 321 (31.9)	41 164 (58.4)	30 048 (53.9)	22 845 (48.3)	<0.001	225 444 (57.9)
Care by cardiologist, *n* (%)	220 208 (92.9)	67 951 (96.1)	52 579 (92.6)	42 969 (86.6)	<0.001	228 093 (58.6)
Optimal care received, *n* (%)	44 530 11.5	18 785 (25.6)	10 992 (18.6)	5958 (11.5)	<0.001	140 895 (36.2)
Percentage of eligible care interventions received, median (IQR)	70.0 (55.6–83.3)	83.3 (66.7–100)	77.8 (63.6–90.0)	72.7 (60.0–87.5)	<0.001	0 (0.0)
Outcomes, *n* (%)						
30-Day mortality	9097 (2.3)	359 (0.5)	1083 (1.8)	2622 (5.0)	<0.001	0 (0.0)
1-Year mortality	55 188 (14.2)	1854 (2.5)	7285 (12.3)	15 616 (30.0)	<0.001	0 (0.0)
8-Year mortality	113 520 (29.2)	3417 (4.7)	12 547 (21.2)	23 578 (45.3)	<0.001	0 (0.0)

ACEi/ARB, angiotensin converting enzyme inhibitor/angiotensin II receptor blocker; COPD, Chronic obstructive pulmonary disease; GRACE, Global Registry Acute Coronary Events; IQR, interquartile range; SD, standard deviation.

a Summary data presented here are based on cases with complete GRACE risk score information only, prior to multiple imputation.

b History of ischaemic heart disease refers to a history of coronary artery bypass grafting, percutaneous coronary intervention, myocardial infarction, or angina.

c Includes numbers and percentage of those eligible.

### Ethical considerations

MINAP is managed by the National Institute for Cardiovascular Outcomes Research (NICOR) (ref. NIGB: ECC 1-06 (d)/2011). NICOR has support under section 251 of the NHS Act 2006 for the conduction of medical research utilizing patient information without formal consent. Formal ethical approval was not required for this study under NHS research governance arrangements for use of non-identifiable patient data. The research complies with the Declaration of Helsinki.

## Results

There were 389 057 patients included in the study with median age of 73 years (IQR 62–81 years) and 143 388 (36.9%) were female (*Figure 1*). There were more low risk [73 351 (39.7%)] than intermediate risk [59 201 (32.1%)] and high risk [52 005 (28.2%)] patients with NSTEMI (*Table 1*). High-risk NSTEMI were older than intermediate and low-risk NSTEMI [84 (79–88) vs. 76 (70–81) vs. 59 (52–66) years, respectively] and more likely to be female (47.9% vs. 40.2% vs. 26.6%). In general, levels of co-morbidity increased with increasing GRACE risk score category (*Table 1*). However, smoking decreased with increasing GRACE risk score category, and a family history of ischaemic heart disease was more frequent in the lower GRACE risk score group. Summary data for patients in whom the GRACE risk score was missing, prior to multiple imputation, is provided in Supplementary material online, *Section S4*, *Table S5*.

In total, 44 530 (11.5%) patients received optimal care, with the median proportion of eligible care received being 70.0% (IQR 55.6–85.7%). Care interventions most frequently not provided were receipt of aldosterone antagonists during admission [9426 (82.5%)], provision of smoking cessation advice [133 726 (80.6)], provision of dietary advice [254 869 (68.1)], and receipt of echocardiogram [181 831 (46.7)]. Both receipt of optimal care and the proportion of care received decreased with increasing GRACE risk score category [optimal care 18 785 (25.6%) and proportion of care 83.3% (IQR 66.7–100) for low-risk NSTEMI vs. 5958 (11.5%) and 72.7% (IQR 60.0–87.5) for high-risk NSTEMI; *P* < 0.001] (*Table 1*, Supplementary material online, *Figure S1*). Receipt of care did not vary significantly by GRACE risk score for electrocardiogram and aspirin on discharge, but increased for in-hospital aldosterone receptor blocker (13.4%, 19.4%, and 20.3% for low-, intermediate-, and high-risk patients, respectively). Whilst receipt of care decreased with increasing GRACE risk score for all other care opportunities, the greatest decreases were observed for coronary angiography (85.4%, 65.7%, and 38%), pre-hospital aspirin (71.1%, 60.0%, and 47.1%), and smoking cessation advice (48.7%, 29.5%, and 18.4%) for low-, intermediate-, and high-risk patients, respectively. Patients with missing GRACE risk score data had similar characteristics to those with complete GRACE risk score data, except for optimal care (8795, 4.3%) and the proportion of care received (63%, IQR 50–75%), which were lower (see Supplementary material online, *Section S4*, *Table S5*).

### Mortality, guideline-indicated treatments, and ischaemic risk

**Figure 2:**
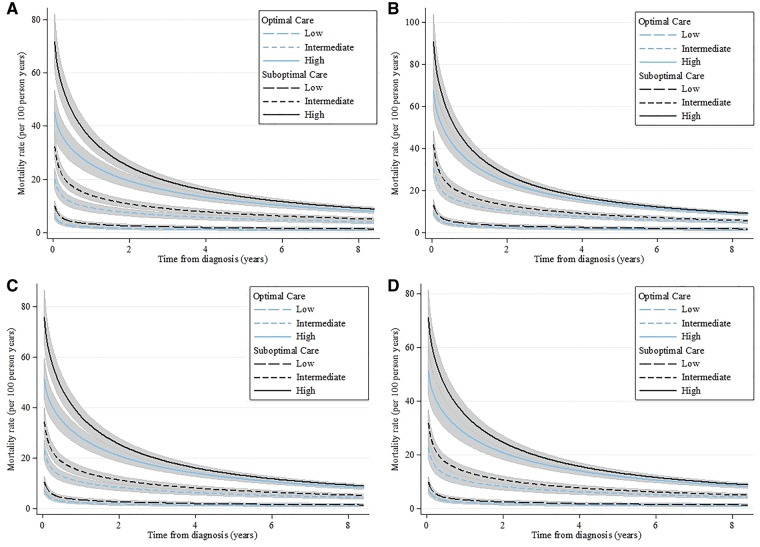
Adjusted* time-varying mortality rates by receipt of optimal care and clinical risk obtained from a flexible parametric model (odds scale, five degrees of freedom) with time varying covariates by GRACE risk score category for optimal care vs. suboptimal care across the full care pathway (*A*) and by the following subgroups of the care pathway: pharmacological therapies (*B*)^†^, investigative and invasive coronary strategies (*C*)^‡^, and lifestyle (D)^§,^^. *P* = 0.004 for interaction. GRACE, Global registry of Acute Coronary Events, categorized into low (<109), intermediate (109 to ≤140), and high (>140) risk. *Model adjusted for demographic characteristics including sex, year, deprivation, previous acute myocardial infarction, previous angina, previous PCI, previous CABG, hypertension, peripheral vascular disease, chronic renal failure, chronic heart failure, cerebrovascular disease, diabetes mellitus, smoking status, and elevated cholesterol. ^†^Including pre-hospital receipt of aspirin, aldosterone antagonist during admission, aspirin on discharge, P2Y_12_ inhibition on discharge, ACE inhibitors (ACEi)/angiotensin receptor blockers (ARBs) on discharge, β-blocker on discharge, and HMG Co-A reductase inhibitor (statin) on discharge. ^‡^Including receipt of a pre- or in-hospital electrocardiogram, echocardiography and coronary angiography. ^§^Including referral for cardiac rehabilitation, smoking cessation advice and dietary advice.

**Figure 3:**
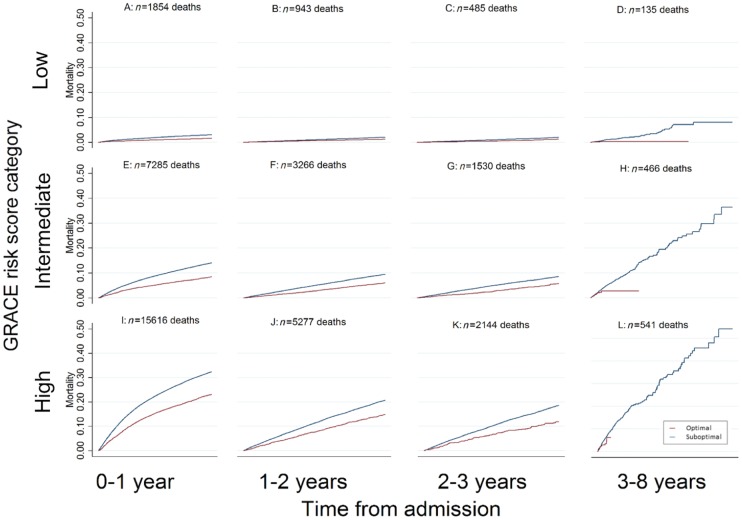
Unadjusted landmark Kaplan–Meier survival curves and crude mortality rates by GRACE risk score category and receipt of optimal care vs. suboptimal care. This figure demonstrates the benchmarked crude mortality rates for the following time periods; 0–1 year, 1–2 years, 2–3 years, 3–8 years, across GRACE risk category by the receipt of optimal care. The percentages represent the crude mortality rates for each time period with the 95% confidence interval for each of the respective GRACE risk score categories. Population at risk at baseline; low GRACE risk category: 73 351, intermediate GRACE risk category: 59 201, high GRACE risk category: 52 005. Population at risk 3–8 years; low GRACE risk category: 70 069 intermediate GRACE risk category: 47 120, high GRACE risk category: 28 698.

There were 113 856 (29.2%) deaths corresponding to 10.5 deaths per 100 person years. A pattern of greater early hazard for death was evident across the spectrum of NSTEMI risk, and accentuated among high-risk NSTEMI (*Figure 2*). Across all GRACE risk score groups for landmark time periods 0–1 years, 1–2 years, and 2–3 years, but not 3–8 years, unadjusted mortality rates were significantly higher for patients who did not receive optimal care (*Figure 3*). After adjustment, there remained a benefit in receiving optimal care regardless of estimated ischaemic risk. [Adjusted hazard ratio (aHR) = 0.62 (95% CI 0.56–0.68) difference in absolute mortality rate per 100 patients (AMR/100) −0.01 (95% CI −0.01 to 0.00) *Table 2*].

**Table 2:** Time-varying adjusted hazard ratios and absolute difference in mortality rate per 100 for patients receiving optimal care compared with suboptimal care after multiple imputation for missing data

	Optimal care vs. suboptimal care	
	aHR^a^	Difference in AMR/100
HR over total follow-up time	0.62 (0.56–0.68)	−0.01 (−0.01 to 0.00)
30 days	0.72 (0.63–0.84)	−0.02 (−0.03 to −0.01)
1	0.57 (0.47–0.73)	−0.01 (−0.02 to −0.01)
2	0.56 (0.44–0.77)	−0.01 (−0.02 to −0.01)
3	0.56 (0.43–0.80)	−0.01 (−0.01 to 0.00)
4	0.56 (0.42–0.82)	−0.01 (−0.01 to 0.00)
5	0.56 (0.42–0.84)	−0.01 (−0.01 to 0.00)
6	0.56 (0.42–0.86)	−0.01 (−0.01 to 0.00)
7	0.56 (0.42–0.87)	−0.01 (−0.01 to 0.00)
8	0.57 (0.42–0.89)	−0.01 (−0.01 to 0.00)

aHR, adjusted hazard ratio; AMR, absolute mortality rate.

^**a**^aHR—adjusted hazard ratio obtained from flexible parametric survival modelling on the odds scale with five degrees of freedom and time-varying covariates for optimal care and GRACE risk, adjusted for: patient demographics (sex, year, and Index of Multiple Deprivation) and medical history (history of diabetes, smoking status, family history of coronary heart disease, hypertension, previous myocardial infarction, previous angina, peripheral vascular disease, cerebrovascular disease, chronic obstructive pulmonary disease or asthma, chronic renal failure, congestive cardiac failure, previous percutaneous coronary intervention, previous coronary artery bypass graft surgery, and total cholesterol).

At 30 days, the use of all eligible guideline-indicated treatments was associated with improved survival among high risk NSTEMI [aHR = 0.66 (95% CI 0.53–0.86) AMR/100 −0.19 (95% CI −0.29 to −0.08)], and intermediate risk NSTEMI [aHR = 0.74 (95% CI 0.62–0.92); AMR/100 −0.15 (95% CI −0.23 to −0.08) *Table 3*]. At the end of follow-up (8.4 years), the significant association between the use of all eligible guideline-indicated treatments and improved survival remained for high-risk NSTEMI (aHR = 0.66, 95% CI 0.50–0.96; AMR/100 = −0.03, 95% CI −0.06 to −0.01), but not for intermediate-risk NSTEMI (aHR = 1.04, 95% CI 0.74–1.71; AMR/100 = 0.002, 95% CI −0.02 to 0.03). For the low-risk NSTEMI, there was no association between use of all compared with the use of some eligible guideline-indicated treatments and improved survival at 30-days (aHR = 0.92, 95% CI 0.69–1.38) and at 8.4 years (aHR = 0.71, 95% CI 0.39–3.74). A sensitivity analysis, which included in-hospital deaths made minimal difference to the effect directions and magnitudes (see Supplementary material online, *Section S5*, *Table S6*).

**Table 3:** Time-varying adjusted hazard ratios and absolute difference in mortality rate per 100 for patients receiving optimal care compared with suboptimal care according to low, intermediate and high GRACE risk score category after multiple imputation for missing data*

	Optimal care vs. suboptimal care		Optimal care vs. suboptimal care		Optimal care vs. suboptimal care	
	Low GRACE risk		Intermediate GRACE risk		High GRACE risk	
	aHR^a^	Difference in AMR/100	aHR^a^	Difference in AMR/100	aHR^a^	Difference in AMR/100
HR over total follow-up time	0.76 (0.60–0.96)	−0.01 (−0.02 to −0.002)	0.66 (0.56–0.77)	−0.03 (−0.04 to −0.02)	0.55 (0.48–0.63)	−0.07 (−0.09 to −0.05)
Time varying HRs						
30 days	0.92 (0.69–1.38)	−0.01 (−0.06 to 0.03)	0.74 (0.62–0.92)	−0.15 (−0.23 to −0.08)	0.66 (0.53–0.86)	−0.19 (−0.29 to −0.08)
1	0.71 (0.47–1.49)	−0.02 (−0.04 to 0.01)	0.85 (0.64–1.25)	−0.03 (−0.08 to 0.02)	0.53 (0.42–0.74)	−0.18 (−0.25 to −0.12)
2	0.71 (0.43–1.93)	−0.01 (−0.04 to 0.01)	0.92 (0.66–1.47)	−0.01 (−0.06 to 0.04)	0.56 (0.42–0.82)	−0.12 (−0.17 to −0.07)
3	0.70 (0.42–2.31)	−0.01 (−0.03 to 0.01)	0.96 (0.68–1.58)	−0.0050 (−0.05 to 0.04)	0.58 (0.44–0.87)	−0.09 (−0.13 to −0.05)
4	0.70 (0.41–2.66)	−0.01 (−0.03 to 0.01)	0.98 (0.70–1.65)	−0.0017 (−0.04 to 0.04)	0.60 (0.45–0.90)	−0.07 (−0.1 to −0.03)
5	0.71 (0.40–2.97)	−0.01 (−0.03 to 0.01)	1.00 (0.71–1.68)	0.0001 (−0.03 to 0.03)	0.62 (0.46–0.92)	−0.06 (−0.09 to −0.02)
6	0.71 (0.40–3.25)	−0.01 (−0.03 to 0.01)	1.02 (0.72–1.70)	0.0012 (−0.03 to 0.03)	0.63 (0.48–0.94)	−0.05 (−0.07 to −0.2)
7	0.71 (0.39–3.51)	−0.01 (−0.02 to 0.01)	1.03 (0.74–1.71)	0.0019 (−0.03 to 0.03)	0.64 (0.49–0.95)	−0.04 (−0.06 to −0.02)
8	0.71 (0.39–3.74)	−0.01 (−0.02 to 0.01)	1.04 (0.74–1.71)	0.0023 (−0.02 to 0.03)	0.66 (0.50–0.96)	−0.03 (−0.06 to −0.01)

aHR, adjusted hazard ratio; AMR, absolute mortality rate; GRACE, Global registry of Acute Coronary Events, categorized into low (<109), intermediate (109 to ≤140), and high (>140) risk.

a aHR—adjusted hazard ratio obtained from flexible parametric survival modelling on the odds scale with five degrees of freedom and time-varying covariates for optimal care and GRACE risk, adjusted for: patient demographics (sex, year, and Index of Multiple Deprivation) and medical history (history of diabetes, smoking status, family history of coronary heart disease, hypertension, previous myocardial infarction, previous angina, peripheral vascular disease, cerebrovascular disease, chronic obstructive pulmonary disease or asthma, chronic renal failure, congestive cardiac failure, previous percutaneous coronary intervention, previous coronary artery bypass graft surgery, and total cholesterol).

* *P* < 0.001 for interaction.

Of the three subgroups of guideline-indicated care treatments and diagnostics, investigative and invasive coronary strategy was associated with the most comprehensive impact on survival—including beneficial effects among low-, intermediate-, and high-risk NSTEMI as well as effects that persisted the longest for the high-risk group (*Figure 2*, Supplementary material online, *Tables S7a–S7c*). For intermediate-risk NSTEMI, investigative and invasive coronary strategies were associated with a 28% relative survival improvement up to 3 years (aHR = 0.72, 95% CI 0.57–0.98; AMR/100 = −0.03, 95% CI −0.06 to −0.01), and for high-risk NSTEMI a 19% survival improvement at 8 years (aHR = 0.81, 95% CI 0.69–0.97; AMR/100 = −0.02, 95% CI −0.03 to −0.01). Pharmacological therapies were associated with a 46% survival improvement at 6 years (aHR = 0.54, 95% CI 0.37–0.97; AMR/100 = −0.02, 95% CI −0.04 to −0.01) for low-risk NSTEMI, and a 25% survival improvement at 5 years (aHR = 0.75, 95% CI 0.61–0.99; AMR/100 = −0.02, 95% CI −0.04 to 0.00) for intermediate-risk NSTEMI, with no persisting effect for high-risk NSTEMI. Lifestyle care opportunities were associated with a 25% survival improvement at 8 years (aHR = 0.75, 95% CI 0.63–0.92; AMR/100 = −0.02, 95% CI −0.04 to −0.01) for high-risk NSTEMI, but no persisting effect for low- or intermediate-risk NSTEMI.

## Discussion

In this prospective observational cohort study of 389 057 patients with NSTEMI using data for all acute hospitals in a single health care system, optimal use of guideline-indicated care for NSTEMI was associated with greater survival gains with increasing GRACE risk, but its use decreased with increasing GRACE risk. Of note, is that the mortality benefit associated with optimal care found in high-risk NSTEMI persisted for over eight years from the time of discharge from hospital. Whilst there was a preponderance of low-risk NSTEMI patients who had high rates of survival, these patients proportionally received more evidence-based care compared with intermediate and high-risk NSTEMI. Moreover, a pattern of early death was evident across the NSTEMI risk spectrum, which was accentuated for those with the highest GRACE risk scores. Taken together, these findings suggest that providing all eligible care opportunities to NSTEMI patients has the potential to improve survival, and that those at highest risk will derive greater and more sustained benefit.

To our knowledge, this is the first study to investigate how long the impact of the pathway of guideline-indicated care according to baseline ischaemic risk among eligible patients with NSTEMI persists. Previous work has demonstrated that guideline-directed therapy results in improved outcomes at 30 days and 3 years, yet is limited because it focuses on the performance of finite quality indicators or interventions rather than cumulative care.^3^^,^^24–26^ This is important because the treatment of NSTEMI follows a journey of care and defined by evidence from randomized controlled trials and observational studies.^14^^,^^15^ Whilst earlier studies have demonstrated excess mortality associated with the non-receipt of guideline indicated interventions along the pathway of care, the potential persistence of effect sizes was not studied.^2^

Low-risk NSTEMI who received all eligible care interventions did not have a significant survival advantage compared with their counterparts who received some or none of the eligible care interventions. Whilst this seems counterintuitive, especially when randomized data have demonstrated clinical benefit from evidence—based treatments for NSTEMI,^27^ there are a number of possible explanations. First, the low GRACE risk score comparator group had high rates of receipt of many care interventions; therefore, although care was not optimal for 54 566 patients in this group, it was still high overall (median receipt of care 83.3%), and indeed, those with a low GRACE risk score comprised the greatest proportion of patients with the highest receipt of care. Second, the low rates of death in the low GRACE risk score group created a ‘floor effect’ whereby the discrimination of differences between optimal and suboptimal care was not possible.

We found that an invasive coronary strategy was associated with the most comprehensive and persistent impact on survival. Such an approach to the treatment of NSTEMI improved survival for intermediate and high-risk patients—with effects lasting for many years after hospital discharge following treatment. We noted that the beneficial survival effect associated with pharmacotherapies was restricted to low- and intermediate-risk NSTEMI for approximately 6 years after hospital discharge, whereas lifestyle modifications were associated with improved survival for up to 8 years among high-risk NSTEMI. We speculate that this differential association with survival may be because the effect of other care interventions such as an invasive strategy is greater than that of pharmacotherapies.^3^ Indeed, the advantages of an invasive strategy on early and mid-term clinical outcomes have been demonstrated in randomized studies,^7^^,^^24^ yet until now the evidence for its impact on longer-term outcomes has been limited.^28^ Our ‘real world’ national study supports these mid-term outcomes data, but also suggests that the impact of coronary angiography and revascularization for NSTEMI extends to at least 8 years.

The utilization of risk scores is recommended by international guidelines.^13–15^ In part, this is because physicians underestimate future ischaemic risk for NSTEMI which in turn contributes to suboptimal use of treatments.^30^^,^^31^ Our research supports the use of accurate risk estimation for NSTEMI, and is in keeping with evidence indicating that the GRACE risk score may be used to predict long-term outcomes.^31–34^ Using a validated risk stratification tool such as the GRACE risk score, may enable earlier mobilization of care interventions, and therefore, reduce fatal and non-fatal cardiovascular events.^35^^,^^36^

Moreover, the international burden of NSTEMI burden is high and is set to increase, with associated high mortality rates in the medium to longer-term.^29^^,^^30^ It is evident that the opportunities to improve care, and therefore realize reductions in cardiovascular endpoints following acute myocardial infarction, are unmet.^1^^,^^2^^,^^25^^,^^36–38^ Given that all of the interventions selected in this study were based on Class 1 recommendations that have been demonstrated to improve outcomes for NSTEMI, a decline in mortality rates should follow an increase in adherence to guideline-indicated care, with greater and persisting benefits among the higher risk.

To our knowledge, MINAP is the largest whole-country, single health system, prospective observational cohort of the quality of care and clinical outcomes across the spectrum of acute coronary syndromes. It is designed to be representative of the management of acute coronary syndrome in a clinical setting and has standardized criteria for defining case mix and treatments. Nevertheless, there were limitations to our study. First, the study was reliant on accurate recording of data, receipt of dietary and smoking advice was low this may be because the receipt of verbal advice is not well recorded. Second, MINAP does not collect all diagnosed cases of NSTEMI within England and Wales, whereby estimates of cases of acute myocardial infarction may be reduced by half compared with a multi-electronic health records approach.^39^ Third, missing data could have biased the estimates. However, an imputation strategy to minimize bias was implemented.^37^^,^^40^ Fourth, it is probable that other factors beyond the hospital stay (such as drug adherence and primary care visits) may also have influenced survival. Fifth, all-cause mortality was studied because cause specific mortality data were not available. This is a limitation because non-cardiovascular deaths may not be attributable to NSTEMI care.^41^ Sixth, in-hospital deaths were excluded, which could have resulted in survivorship bias—even so, a sensitivity analysis revealed that exclusion of these cases did not affect the conclusions drawn. Seventh is that the non-receipt of guideline care treatment and diagnostics may be a marker of higher NSTEMI risk. Indeed, we found that higher attainment of care occurred for lower risk NSTEMI. Finally, this observational study cannot demonstrate causation.

## Conclusion

For nearly 400 000 NSTEMI hospital survivors in England and Wales, guideline-indicated treatment was less frequent among the high-risk NSTEMI, but when provided was significantly associated with improved survival which persisted over the longer-term. There was benefit seen in those at lower risk, though this was not significant over the whole study period. The provision of ‘up to standard’ guideline-indicated care for high-risk NSTEMI has the potential to improve their longer-term survival.

## Supplementary Material

Supplementary Data
