# Supplementary material for: Guideline-indicated treatments and diagnostics, GRACE risk score, and survival for non-ST elevation myocardial infarction
Source: Eur Heart J. 2018 Sep 7;39(42):3798–806. doi: 10.1093/eurheartj/ehy517 (PMC6220125; doi:10.1093/eurheartj/ehy517)
Supplement: Supplementary Data [file ehy517_online_supplementary_material.docx]

**Online Supplementary Material**

**Title:** Improved survival is associated with guideline-indicated treatments which persists and is greater for higher risk non-ST-elevation myocardial infarction

**eSection 1: Guideline recommended care interventions**

eTable 1: Guideline recommended care interventions and eligibility criteria

**eSection 2: Model selection**

eTable 2: Bayes information criterion (BIC) according to varying degrees of freedom (one to five) and scale (hazard, odds and normal) for adjusted flexible parametric survival model

**eSection 3: Multiple imputation specification and sensitivity analysis**

eTable 3: Imputation model specification.

eTable 4: Adjusted hazard ratios (aHR) for patients receiving optimal care compared with suboptimal care according to low, intermediate and high GRACE risk. Complete case sensitivity analyses.

**eSection 4: Patient characteristics**

eTable 5: Baseline characteristics and care interventions received by GRACE risk score category

eFigure 1: Percentage of low, intermediate and high risk NSTEMI per quartile of receipt of care proportion

**eSection 5: Cohort choice sensitivity analysis**

eTable 6: Adjusted hazard ratios (aHR) for patients receiving optimal care compared with non-optimal care according to low, intermediate and high GRACE risk including patients who died in hospital (n=419,350).

eTable 7: Adjusted hazard ratios (aHR) for patients receiving optimal care compared with non-optimal care according to low, intermediate and high GRACE risk including patients who died in hospital (n=419,350).

**eSection 6: Subgroup analyses of the care pathway**

eTable 8a: Time-varying adjusted hazard ratios (aHR) and absolute difference in mortality rate (AMR) per 100 for patients receiving optimal care compared with suboptimal care for investigate and invasive coronary strategies^*^ according to low, intermediate and high GRACE risk after multiple imputation for missing data.

eTable 8b: Time-varying adjusted hazard ratios (aHR) and absolute difference in mortality rate (AMR) per 100 for patients receiving optimal care compared with suboptimal care of pharmacological therapies^*^ according to low, intermediate and high GRACE risk after multiple imputation for missing data.

eTable 8c: Time-varying adjusted hazard ratios (aHR) and absolute difference in mortality rate (AMR) per 100 for patients receiving optimal care compared with suboptimal care of lifestyle care opportunities^*^ according to low, intermediate and high GRACE risk after multiple imputation for missing data.

**eSection 1: Guideline recommended care interventions**

The care interventions used within the study were determined following review of international guidelines for non-ST-elevation myocardial infarction^1-3^ and mapped to MINAP data, following previously published work by the authors.^4, 5^ Patients were deemed eligible for a care intervention unless there was a contradictory statement, for example if a patient refused a care intervention, or were listed as contraindicated. For certain care interventions patients had to fulfil specific requirements, as detailed in eTable 1 below.

**eTable 1: Guideline recommended care interventions and eligibility criteria**

| **Care intervention** | **Eligibility criteria** |
| --- | --- |
| ECG during admission | All patients |
| Receipt of prehospital aspirin | All patients admitted by the ambulance service, not already on aspirin and in whom aspirin was recorded as not contraindicated. Between years 2003-13 |
| Echocardiogram | All patients unless listed as not indicated. Between years 2003-13. |
| Receipt of angiography | All patients unless there was a contradictory piece of information from the reason why no angiogram was performed data field. Between years 2003-13 |
| Aspirin on discharge | All patients unless listed as “contraindicated / patient declined treatment / not applicable /not indicated”. Between years 2003-13 |
| P2Y_12_ inhibition on discharge | All patients unless listed as “contraindicated / patient declined treatment / not applicable /not indicated”. Between years 2003-13 |
| ACEi/ARB on discharge | All patients unless listed as “contraindicated / patient declined treatment / not applicable /not indicated” between 2003 and 2006.  All patients with LVEF <0.4, or evidence of heart failure or evidence of diabetes and not listed as “contraindicated / patient declined treatment / not applicable /not indicated” between 2007 and 2013. |
| β-Blocker on discharge | All patients unless listed as “contraindicated / patient declined treatment / not applicable /not indicated” between 2003 and 2011.  Ejection fraction of <0.4 and not listed as “contraindicated / patient declined treatment / not applicable /not indicated” between 2011 and 2013. |
| Receipt of aldosterone antagonist during admission | Ejection fraction of <0.4 and one of diabetes or evidence of heart failure. Treated with β-blocker and ACEi/ARB and no evidence of chronic renal failure. Between years 2007-13 |
| HMG Co-A reductase inhibitor (statin) on discharge | All patients unless listed as “contraindicated / patient declined treatment / not applicable /not indicated”. Between years 2003-13 |
| Referral for cardiac rehabilitation | All patients unless listed as not indicated or patient declined. Between years 2003-13 |
| Receipt of smoking cessation advice | Current or previous smoker and not listed as not applicable. Between years 2003-13 |
| Receipt of dietary advice | All patients unless listed as not applicable. Between years 2003-13 |
| Care by cardiologist | All patients |
| Optimal care received | All patients, calculated as an all or none variable. Between years 2003-13 |

**eSection 2: Model selection**

**eTable 2: Bayes information criterion (BIC) according to varying degrees of freedom (one to five) and scale (hazard, odds and normal) for adjusted flexible parametric survival model**

| **Degrees of Freedom** | **Scale** | | |
| --- | --- | --- | --- |
|  | **Hazard** | **Odds** | **Normal** |
| **Model without time varying covariates^*^** | | | |
| 1 | 245811.40 | 245645.07 | 247755.43 |
| 2 | 245564.84 | 245642.98 | 246607.09 |
| 3 | 245510.63 | 245511.11 | 246600.1 |
| 4 | 245413.86 | 245388.88 | - |
| 5 | 245285.87 | **245280.30^$^** | - |
| **Model with time varying GRACE risk and optimal care^*^** | | | |
| 1 | 245529.34 | 245626.08 | 245927.75 |
| 2 | 245537.37 | 245635.57 | 245904.96 |
| 3 | 245475.92 | 245489.52 | 245881.97 |
| 4 | 245390.7 | 245378.13 | - |
| 5 | 245267.66 | **245273.51^$^** | - |
| Abbreviations: GRACE – Global registry of Acute Coronary Events;  **^*^**Adjusted for: Optimal care, GRACE risk score, patient demographics (sex, year, Index of Multiple Deprivation) and medical history (history of diabetes, smoking status, family history of coronary heart disease, hypertension, previous myocardial infarction, previous angina, peripheral vascular disease, cerebrovascular disease, chronic obstructive pulmonary disease or asthma, chronic renal failure, congestive cardiac failure, previous percutaneous coronary intervention, previous coronary artery bypass graft surgery, total cholesterol).  - Model failed to convert at these specifications.  ^$^Optimal model fit based on minimisation of BIC | | | |

**eSection 3: Multiple imputation specification and sensitivity analysis**

Multiple imputation by chained equations (MICE) was used to generate 10 imputed datasets using 20 iterations. The imputation strategy details are provided in eTable 3. As per multiple imputation good practice guidelines,^6^ the main analysis results (manuscript Table 2), which accounted for missing data using multiple imputation, were compared to a complete case analysis (eTable 4).

The analytical cohort for the complete case analysis included only those patients for which complete data was available for each variable included in the model (n=184,390 and 39,513 deaths), which compared to the full analytical cohort of 389,057 patients and 113,586 deaths included in the multiple imputation analysis. The complete case analyses estimates in eTable 4 were consistent with the multiple imputation analyses in manuscript Table 2.

**eTable 3: Imputation model specification.**

| **Variable** | **Variable Type** | **Imputation method** |
| --- | --- | --- |
| Age | Continuous, non-normal | Predictive mean matching |
| Index of multiple deprivation score | Continuous, non-normal | Predictive mean matching |
| Ethnicity | Categorical | Polytomous logistic regression |
| Sex | Binary | Logistic regression |
| Systolic blood pressure | Continuous, non-normal | Predictive mean matching |
| Heart rate | Continuous, non-normal | Predictive mean matching |
| Peak troponin | Continuous, non-normal | Predictive mean matching |
| Creatinine | Continuous, non-normal | Predictive mean matching |
| Loop diuretic | Binary | Logistic regression |
| Cardiac arrest | Binary | Logistic regression |
| ECG Appearance | Categorical | Polytomous logistic regression |
| Aspirin | Binary | Logistic regression |
| Beta-blocker | Binary | Logistic regression |
| Statin | Binary | Logistic regression |
| ACEi/ARBs | Binary | Logistic regression |
| P2Y_12_ inhibitors | Binary | Logistic regression |
| Aldosterone antagonist | Binary | Logistic regression |
| Coronary intervention | Categorical | Polytomous logistic regression |
| Diabetes Mellitus | Binary | Default imputation (missing set to no). |
| Previous hypertension | Binary | Default imputation (missing set to no). |
| Previous hypercholesterolaemia | Binary | Default imputation (missing set to no). |
| Previous MI | Binary | Default imputation (missing set to no). |
| Previous angina | Binary | Default imputation (missing set to no). |
| Peripheral vascular disease | Binary | Default imputation (missing set to no). |
| Cerebrovascular disease | Binary | Default imputation (missing set to no). |
| Chronic obstructive pulmonary disease or asthma | Binary | Default imputation (missing set to no). |
| Congestive renal failure | Binary | Default imputation (missing set to no). |
| Congestive cardiac failure | Binary | Default imputation (missing set to no). |
| Previous PCI | Binary | Default imputation (missing set to no). |
| Previous CABG | Binary | Default imputation (missing set to no). |
| Family history of chronic heart disease | Binary | Default imputation (missing set to no). |
| Smoking status | Binary | Default imputation (missing set to no). |
| Care by a cardiologist | Binary | Default imputation (missing set to no). |
| GRACE Score by time interaction | Continuous, Non-normal | Predictive mean matching |
| Optimal care by time interaction | Continuous, Non-normal | Predictive mean matching |
| Year | Continuous | Predictor variable only |
| Admission diagnosis | Categorical | Predictor variable only |
| Timing of invasive strategy | Continuous | Predictor variable only |
| Nelson-Aalen estimate of survival | Continuous | Predictor variable only |
| Censoring indicator | Binary | Predictor variable only |

**eTable 4: Adjusted hazard ratios (aHR) for patients receiving optimal care compared with suboptimal care according to low, intermediate and high GRACE risk. Complete case sensitivity analyses.**

|  | **Optimal care vs. suboptimal care**  **Low GRACE risk**  **(n=38,133)** | **Optimal care vs. suboptimal care**  **Intermediate GRACE risk**  **(n=52,101)** | **Optimal care vs. suboptimal care**  **High GRACE risk**  **(n=94,156)** |
| --- | --- | --- | --- |
|  | **aHR** | **aHR** | **aHR** |
| **HR over total follow-up time** | 0.71 (0.59-0.86) | 0.67 (0.61-0.73) | 0.54 (0.51-0.56) |
|  | | | |
| 30 days | 0.59 (0.41-0.83) | 0.61 (0.53-0.71) | 0.57 (0.53-0.61) |
| 1 | 0.76 (0.63-0.93) | 0.71 (0.65-0.78) | 0.61 (0.59-0.64) |
| 2 | 0.84 (0.64-1.10) | 0.74 (0.65-0.84) | 0.62 (0.58-0.66) |
| 3 | 0.89 (0.63-1.27) | 0.75 (0.65-0.88) | 0.64 (0.59-0.68) |
| 4 | 0.93 (0.62-1.40) | 0.77 (0.65-0.91) | 0.66 (0.61-0.71) |
| 5 | 0.96 (0.61-1.51) | 0.78 (0.65-0.94) | 0.67 (0.62-0.73) |
| 6 | 0.99 (0.61-1.60) | 0.80 (0.65-0.97) | 0.69 (0.63-0.74) |
| 7 | 1.01 (0.60-1.68) | 0.81 (0.66-0.99) | 0.70 (0.65-0.76) |
| 8 | 1.02 (0.60-1.74) | 0.82 (0.66-1.01) | 0.71 (0.66-0.77) |
| ^*^Abbreviations: GRACE – Global registry of Acute Coronary Events, categorised into low (<109), intermediate (109 to <140) and high (>140) risk; aHR – adjusted hazard ratio obtained from flexible parametric survival modelling on the odds scale with 2 degrees of freedom and time-varying covariates for optimal care and GRACE risk, adjusted for: patient demographics (sex, year, Index of Multiple Deprivation) and medical history (history of diabetes, smoking status, family history of coronary heart disease, hypertension, previous myocardial infarction, previous angina, peripheral vascular disease, cerebrovascular disease, chronic obstructive pulmonary disease or asthma, chronic renal failure, congestive cardiac failure, previous percutaneous coronary intervention, previous coronary artery bypass graft surgery, total cholesterol). | | | |

**eSection 4: Patient characteristics**

**eTable 5: Baseline characteristics and care interventions received by GRACE risk score category**

|  | **Analytical cohort**  **n=389,057** | **Grace risk score category**  **n=184,557** | | | **P value** | **Missing GRACE risk score**  **n=204,500** | **Missing data**  **(n, % of analytical cohort)** |
| --- | --- | --- | --- | --- | --- | --- | --- |
|  |  | **Low (<109)**  **n=73,351 (39.7%)** | **Intermediate (109 to <140)**  **n=59,201 (32.1%)** | **High (>140)**  **n=52,005 (28.2%)** |  |  |  |
| **Patient demographics** |  |  |  |  |  |  |  |
| Age, median, Inter Quartile Range (IQR), years | 72.7 (61.7-81.2) | 59.5 (52.0 – 66.0) | 76.0 (70.4 – 81.0) | 84.0 (79.0 – 88.0) | <.001 | 72.5 (61.7-81.0) | 638 (0.2) |
| Sex (N, % males) | 244,837 (63.1) | 53,818 (73.4) | 35,442 (59.9) | 27,104 (52.1) | <.001 | 128,473 (63.0) | 258 (0.1) |
| **Patient medical History and clinical measures** |  |  |  |  |  |  |  |
| History of Ischaemic Heart Disease* (n, %) | 162,064 (45.2) | 22,885 (31.4) | 29,334 (50.0) | 28,676 (55.7) | <.001 | 81,169 (46.3) | 23,879 (6.1) |
| Hypertension (n, %) | 188,503 (48.5) | 33,872 (46.5) | 94,894 (59.4) | 30,605 (59.5) | <.001 | 89,132 (51.3) | 25,991 (6.7) |
| Diabetes (n, %) | 81,469 (20.9) | 13,229 (18.2) | 15,771 (26.9) | 13,598 (26.5) | <.001 | 38,871 (21.8) | 27,712 (7.1) |
| Dyslipidaemia (n, %) | 121,243 (33.7) | 27,292 (38.0) | 21,893 (37.9) | 15,952 (31.6) | <.001 | 56,106 (33.7) | 28,771 (7.4) |
| Family history of IHD (n, %) | 77,288 (26.2) | 29,184 (44.0) | 12,302 (25.0) | 5,915 (14.8) | <.001 | 29,887 (33.7) | 94,215 (24.2) |
| Smoking status (current or previous smoker vs. never smoked) (n, %) | 217,116 (60.3) | 49,323 (68.6) | 33,327 (59.0) | 25,589 (53.1) | <.001 | 108,877 (59.4) | 29,219 (7.5) |
| Peripheral vascular disease (n, %) | 18,324 (5.2) | 2,181 (3.1) | 3,431 (6.0) | 3,301 (6.5) | <.001 | 9,411 (5.7) | 34,467 (8.9) |
| Congestive cardiac failure (n, %) | 24,529 (6.9) | 1,205 (1.7) | 3,759 (6.4) | 7,683 (15.0) | <.001 | 11,882 (7.2) | 33,304 (8.6) |
| COPD or asthma (n, %) | 56,708 (14.6) | 9,176 (12.8) | 10,796 (18.6) | 10,176 (20.1) | <.001 | 26,560 (16.2) | 33,633 (8.6) |
| Chronic kidney disease (n, %) | 21,938 (6.2) | 1,637 (2.3) | 4,349 (7.4) | 7,216 (14.1) | <.001 | 8,736 (5.3) | 33,448 (8.6) |
| Cerebrovascular disease (n, %) | 34,146 (9.6) | 3,505 (4.8) | 7,146 (12.2) | 7,867 (15.3) | <.001 | 15,628 (9.5) | 34,302 (8.8) |
| Heart rate (median IQR), bpm | 80 (67 -95) | 74.0 (64.0 – 86.0) | 79.0 (66.0 – 92.0) | 89.0 (74.0 – 107.0) | <.001 | 80 (67 - 96) | 65,863 (16.9) |
| Systolic Blood Pressure (mean, SD), mmHg | 142.5 (28.4) | 149.1 (26.5) | 144.3 (27.3) | 131.0 (27.0) | <.001 | 142.7 (29.1) | 66,688 (17.1) |
| Cardiac Arrest (pre-hospital), (n, %) | 1,305 (0.7%) | 99 (0.1) | 354 (0.6) | 852 (1.6) | <.001 | 1,307 (0.6) | 22,901 (5.9) |
| Initial Creatinine (median IQR), µmol/L | 92.0 (76.0-114.0) | 84.0  (72.0 – 98.0) | 94.0  (78.0 – 117.0) | 110.0  (86.0 – 144.0) | <.001 | 91 (76.0-113.0) | 165,622 (42.6) |
| Peak Troponin, (median IQR), ng/ml | 1200 (260 -815) | 1200  (210 – 12,000) | 1300  (240 – 13,900) | 1860  (340 – 17,210) | <.001 | 1060 (260-5460) | 19,114 (4.9) |
| ST deviation on admission, (n, %) | 108,189 (30.62) | 12,117 (16.5) | 16,262 (27.5) | 22,720 (43.7) | <.001 | 57,090 (33.8) | 35,699 (9.2) |
| **Care interventions** |  |  |  |  |  |  |  |
| ECG during admission, (n, %) | 371,149 (95.4) | 73,351 (100) | 59,201 (100) | 52,005 (100) | NS | 186,592 (91.2) | 9295 (4.7) |
| Receipt of prehospital aspirin†, (n, %) | 91,679 (70.8) | 21,681 (71.1) | 13,915 (60.0) | 8,721 (47.1) | <.001 | 47,362 (82.8) |  |
| Echocardiogram†, (n, %) | 207,128 (53.3) | 44,772 (61.0) | 36,834 (62.2) | 32,404 (62.3) | <.001 | 93,188 (45.6) | 11,053 (2.8) |
| Receipt of angiography†, (n, %) | 198,303 (55.7) | 60,063 (85.4) | 34,691 (65.7) | 15,903 (38.0) | <.001 | 87,646 (46.0) | 15,656 (4.0) |
| Aspirin on discharge†, (n, %) | 301,639 (88.46) | 56,130 (92.2) | 45,626 (92.6) | 39,458 (92.1) | 0.95 | 160,425 (85.4) | 32,983 (8.5) |
| P2Y_12_ inhibition on discharge†, (n, %) | 127,315 (93.1) | 39,858 (95.7) | 31,105 (93.1) | 24,867 (89.3) | <.001 | 31,485 (93.0) | 4,236 (1.1) |
| ACEi/ARB on discharge†, (n %) | 169,942 (78.9) | 15,400 (91.5) | 21,360 (89.9) | 26,715 (86.2) | <.001 | 106,467 (74.1) | 206,719 (53.1) |
| β-Blocker on discharge†, (n, %) | 138,656 (78.8) | 7,625 (92.3) | 14,730 (90.5) | 24,953 (88.4) | <.001 | 91,348 (74.1) | 5,300 (1.4) |
| Receipt of aldosterone antagonist during admission†, (n, %) | 2,004 (17.5) | 191 (13.4) | 614 (19.4) | 990 (20.3) | <.001 | 209 (10.7) | 4,798 (1.2) |
| Statin on discharge†, (n, %) | 297,045 (85.4) | 55,965 (91.4) | 45,909 (90.8) | 38,916 (87.4) | <.001 | 156,255 (81.6) |  |
| Referral for cardiac rehabilitation†, (n, %) | 279,027 (76.0) | 60,450 (86.1) | 44,508 (81.5) | 33,671 (74.6) | <.001 | 140,398 (71.3) | 4,519 (1.2) |
| Smoking cessation advice received†, (n, %) | 32,109 (19.4) | 17,405 (48.7) | 5,434 (29.47) | 2,350 (18.4) | <.001 | 6,920 (7.0) | 11,658 (3.0) |
| Dietary advice received†, (n, %) | 119,321 (31.9) | 41,164 (58.4) | 30,048 (53.9) | 22,845 (48.3) | <.001 | 25,264 (12.6) | 225,444 (57.9) |
| Care by cardiologist, (n, %) | 220,208 (92.9) | 67,951 (96.1) | 52,579 (92.6) | 42,969 (86.6) | <.001 | 56,709 (94.5) | 228,093 (58.6) |
| Optimal care received | 44,530 11.5 | 18,785 (25.6) | 10,992 (18.6) | 5,958 (11.5) | <.001 | 8,795 (4.3) | 140,895 (36.2) |
| Percentage of eligible care interventions received (median, IQR) | 70.0 (55.6 - 83.3) | 83.3 (66.7 – 100) | 77.8 (63.6 – 90.0) | 72.7 (60.0 -87.5) | <.001 | 63.6 (50.0 – 75.0) | 0 (0.0) |
| Mortality (post discharge) (n, %) | 113,856 (29.2) | 3,417 (4.7) | 12,547 (21.2) | 23,578 (45.3) | <.001 | 74,044 (36.2) | 0 (0.0) |

*History of ischaemic heart disease refers to a history of CABG, MI, PCI or angina

†Includes numbers and % of those eligible

*Abbreviations*

SD; standard deviation, IHD; ischaemic heart disease, COPD; Chronic obstructive pulmonary disease, IQR; interquartile range, GRACE; Global Registry Acute Coronary Events, ACEi/ARB; Angiotensin converting enzyme inhibitor / Angiotensin II receptor blocker, PCI; Percutaneous Coronary Intervention, CABG; Coronary Artery Bypass Grafting.

**eFigure 1: Percentage of low, intermediate and high risk NSTEMI per quartile of receipt of care proportion**


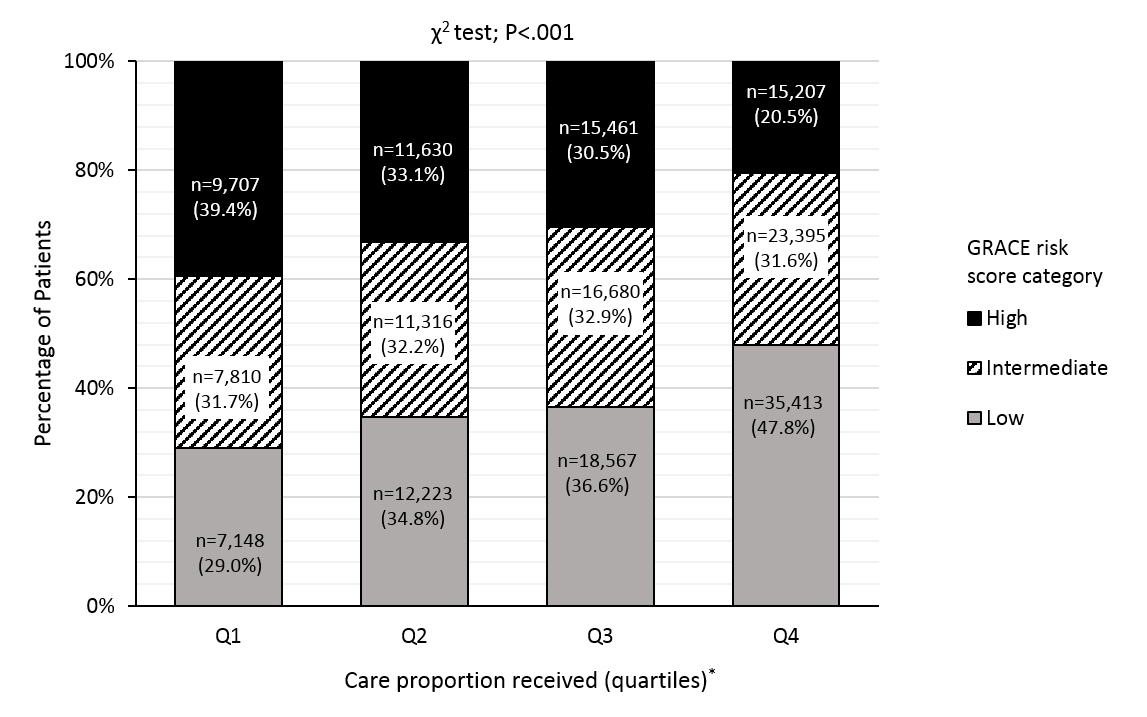


^*^Quartiles represent the following care proportions; Q1: 0-0.54, Q2: 0.55-0.69, Q3: 0.70-0.85, Q4: 0.86-1.00.

**eSection 5: Cohort choice sensitivity analysis**

|  | **Optimal care vs. non-optimal care**  **Low GRACE risk**  **(n=78,079)** | **Optimal care vs. non-optimal care**  **Intermediate GRACE risk**  **(n=** **67,924)** | **Optimal care vs. non-optimal care**  **High GRACE risk**  **(n=** **36,586)** |
| --- | --- | --- | --- |
|  | **aHR** | **aHR** | **aHR** |
| **HR over total follow-up time** | 0.63 (0.58-0.68) | 0.50 (0.47-0.52) | 0.43 (0.39-0.45) |
| 30 days | 0.82 (0.73-0.93) | 0.87 (0.62-1.21) | 0.56 (0.51-0.61) |
| 1 | 0.91 (0.84-0.99) | 0.90 (0.85-0.96) | 0.45 (0.41-0.49) |
| 2 | 0.94 (0.86-1.04) | 0.91 (0.86-0.97) | 0.43 (0.39-0.47) |
| 3 | 0.96 (0.87-1.06) | 0.92 (0.86-0.98) | 0.41 (0.37-0.46) |
| 4 | 0.96 (0.87-1.07) | 0.92 (0.86-0.98) | 0.40 (0.36-0.46) |
| 5 | 0.97 (0.87-1.07) | 0.92 (0.86-0.98) | 0.40 (0.35-0.45) |
| 6 | 0.97 (0.87-1.07) | 0.92 (0.86-0.99) | 0.39 (0.34-0.45) |
| 7 | 0.97 (0.87-1.07) | 0.92 (0.86-0.99) | 0.39 (0.33-0.45) |
| 8 | 0.97 (0.87-1.07) | 0.92 (0.86-0.99) | 0.38 (0.33-0.45) |
| ^*^Abbreviations: GRACE – Global registry of Acute Coronary Events, categorised into low (<109), intermediate (109 to <140) and high (>140) risk; aHR – adjusted hazard ratio obtained from flexible parametric survival modelling on the odds scale with 2 degrees of freedom and time-varying covariates for optimal care and GRACE risk, adjusted for: patient demographics (sex, year, Index of Multiple Deprivation) and medical history (history of diabetes, smoking status, family history of coronary heart disease, hypertension, previous myocardial infarction, previous angina, peripheral vascular disease, cerebrovascular disease, chronic obstructive pulmonary disease or asthma, chronic renal failure, congestive cardiac failure, previous percutaneous coronary intervention, previous coronary artery bypass graft surgery, total cholesterol). | | | |

**eTable 6: Adjusted hazard ratios (aHR) for patients receiving optimal care compared with non-optimal care according to low, intermediate and high GRACE risk including patients who died in hospital (n=419,350).**

**eTable 7: Adjusted hazard ratios (aHR) for patients receiving optimal care compared with non-optimal care according to low, intermediate and high GRACE risk including patients who died in hospital (n=419,350).**

|  | **Standard Cox Regression Model**  **HR (95% CI)** | **Cox regression model including shared frailty to account for hospital level clustering**  **HR (95% CI)** | **Hospital level variance (SD)** |
| --- | --- | --- | --- |
| **Optimal vs. suboptimal care** |  |  |  |
| Overall | 0.637 (0.582-0.697); p<0.001 | 0.639 (0.582-0.702); p<0.001 | 0.031 (0.004); P<0.001 |
| GRACE risk stratified |  |  |  |
| Low | 0.764 (0.605-0.965); p=0.024 | 0.790 (0.621-1.000); p=0.05 | 0.048 (0.026); p=0.012 |
| Intermediate | 0.675 (0.581-0.783); p<0.001 | 0.677 (0.58-0.79); p<0.001 | 0.047 (0.010); p<0.001 |
| High | 0.593 (0.520-0.676); p<0.001 | 0.576 (0.505-0.659); p<0.001 | 0.030 (0.004); p<0.001 |

**eSection 6: Subgroup analyses of the care pathway**

**eTable 8a:** **Time-varying adjusted hazard ratios (aHR) and absolute difference in mortality rate (AMR) per 100 for patients receiving optimal care compared with suboptimal care for investigate and invasive coronary strategies^*^ according to low, intermediate and high GRACE risk after multiple imputation for missing data.**

|  | **Optimal care vs. suboptimal care**  **Low GRACE risk** | | **Optimal care vs. suboptimal care**  **Intermediate GRACE risk** | | **Optimal care vs. suboptimal care**  **High GRACE risk** | |
| --- | --- | --- | --- | --- | --- | --- |
|  | **aHR** | **Difference in AMR/100** | **aHR** | **Difference in AMR/100** | **aHR** | **Difference in AMR/100** |
| **HR over total follow-up time** | 0.78 (0.67-0.91) | -0.01 (-0.01 to 0.00) | 0.64 (0.71-0.57) | -0.03 (-0.04 to -0.03) | 0.61 (0.56-0.67) | -0.06 (-0.07 to -0.05) |
| **Time varying HR at set follow-up times** | | | | | | |
| 30 days | 0.70 (0.58-0.88) | -0.05 (-0.08 to -0.02) | 0.72 (0.63-0.84) | -0.13 (-0.18 to -0.07) | 0.74 (0.66-0.84) | -0.19 (-0.26 to -0.12) |
| 1 year | 1.01 (0.76-1.48) | 0.00 (-0.02 to 0.02) | 0.68 (0.56-0.86) | -0.06 (-0.10 to -0.03) | 0.66 (0.57-0.80) | -0.14 (-0.19 to -0.09) |
| 2 years | 1.09 (0.78-1.78) | 0.00 (-0.01 to 0.02) | 0.70 (0.56-0.94) | -0.04 (-0.07 to -0.01) | 0.70 (0.59-0.86) | -0.08 (-0.12 to -0.04) |
| 3 years | 1.13 (0.79-1.97) | 0.01 (-0.01 to 0.02) | 0.72 (0.57-0.98) | -0.03 (-0.06 to -0.01) | 0.73 (0.62-0.90) | -0.06 (-0.09 to -0.03) |
| 4 years | 1.16 (0.80-2.10) | 0.01 (-0.01 to 0.02) | 0.74 (0.58-1.01) | -0.03 (-0.05 to 0.00) | 0.75 (0.64-0.92) | -0.04 (-0.07 to -0.02) |
| 5 years | 1.19 (0.81-2.20) | 0.01 (-0.01 to 0.02) | 0.75 (0.59-1.04) | -0.02 (-0.04 to 0.00) | 0.77 (0.65-0.94) | -0.03 (-0.05 to -0.01) |
| 6 years | 1.20 (0.82-2.27) | 0.01 (-0.01 to 0.02) | 0.77 (0.60-1.05) | -0.02 (-0.04 to 0.00) | 0.78 (0.67-0.95) | -0.03 (-0.05 to -0.01) |
| 7 years | 1.22 (0.83-2.33) | 0.01 (-0.01 to 0.02) | 0.78 (0.61-1.07) | -0.02 (-0.03 to 0.00) | 0.80 (0.68-0.96) | -0.02 (-0.04 to -0.01) |
| 8 years | 1.23 (0.83-2.37) | 0.01 (-0.01 to 0.02) | 0.79 (0.62-1.08) | -0.01 (-0.03 to 0.00) | 0.81 (0.69-0.97) | -0.02 (-0.03 to -0.01) |
| ^*^Including receipt of a pre- or in-hospital electrocardiogram, echocardiography and coronary angiography | | | | | | |

**eTable 8b:** **Time-varying adjusted hazard ratios (aHR) and absolute difference in mortality rate (AMR) per 100 for patients receiving optimal care compared with suboptimal care of pharmacological therapies^*^ according to low, intermediate and high GRACE risk after multiple imputation for missing data.**

|  | **Optimal care vs. suboptimal care**  **Low GRACE risk** | | **Optimal care vs. suboptimal care**  **Intermediate GRACE risk** | | **Optimal care vs. suboptimal care**  **High GRACE risk** | |
| --- | --- | --- | --- | --- | --- | --- |
|  | **aHR** | **Difference in AMR/100** | **aHR** | **Difference in AMR/100** | **aHR** | **Difference in AMR/100** |
| **HR over total follow-up time** | 0.58 (0.50-0.67) | -0.02 (-0.03 to -0.01) | 0.69 (0.63-0.76) | -0.03 (-0.04 to -0.02) | 0.85 (0.78-0.92) | -0.02 (-0.04 to 0.01) |
| **Time varying HR at set follow-up times** | | | | | | |
| 30 days | 0.65 (0.54-0.81) | -0.07 (-0.11 to -0.03) | 0.83 (0.73-0.96) | -0.09 (-0.16 to -0.02) | 0.94 (0.85-1.05) | -0.05 (-0.14 to 0.03) |
| 1 year | 0.53 (0.40-0.77) | -0.04 (-0.07 to -0.02) | 0.70 (0.58-0.87) | -0.07 (-0.11 to -0.03) | 0.86 (0.76-1.00) | -0.06 (-0.12 to 0.00) |
| 2 years | 0.53 (0.38-0.85) | -0.03 (-0.06 to -0.02) | 0.72 (0.58-0.92) | -0.05 (-0.08 to -0.02) | 0.88 (0.77-1.03) | -0.04 (-0.08 to 0.00) |
| 3 years | 0.53 (0.37-0.90) | -0.03 (-0.05 to -0.02) | 0.73 (0.59-0.95) | -0.04 (-0.06 to -0.01) | 0.89 (0.78-1.04) | -0.02 (-0.06 to 0.01) |
| 4 years | 0.53 (0.37-0.94) | -0.02 (-0.04 to -0.02) | 0.74 (0.60-0.97) | -0.03 (-0.05 to -0.01) | 0.90 (0.79-1.05) | -0.02 (-0.04 to 0.01) |
| 5 years | 0.54 (0.37-0.97) | -0.02 (-0.04 to -0.01) | 0.75 (0.61-0.99) | -0.02 (-0.04 to 0.00) | 0.91 (0.80-1.05) | -0.01 (-0.03 to 0.01) |
| 6 years | 0.54 (0.37-0.99) | -0.02 (-0.04 to -0.01) | 0.76 (0.62-1.00) | -0.02 (-0.04 to 0.00) | 0.91 (0.80-1.05) | -0.01 (-0.03 to 0.01) |
| 7 years | 0.54 (0.37-1.01) | -0.02 (-0.03 to -0.01) | 0.77 (0.63-1.01) | -0.02 (-0.03 to 0.00) | 0.92 (0.81-1.05) | -0.01 (-0.02 to 0.00) |
| 8 years | 0.55 (0.37-1.03) | -0.02 (-0.03 to -0.01) | 0.78 (0.63-1.01) | -0.02 (-0.03 to 0.00) | 0.92 (0.81-1.06) | -0.01 (-0.02 to 0.00) |
| ^*^Including pre-hospital receipt of aspirin, aldosterone antagonist during admission, aspirin on discharge, P2Y12 inhibition on discharge, ACE inhibitors (ACEi)/Angiotensin receptor blockers (ARBs) on discharge, β-blocker on discharge and HMG Co-A reductase inhibitor (statin) on discharge. | | | | | | |

**eTable 8c:** **Time-varying adjusted hazard ratios (aHR) and absolute difference in mortality rate (AMR) per 100 for patients receiving optimal care compared with suboptimal care of lifestyle care opportunities^*^ according to low, intermediate and high GRACE risk after multiple imputation for missing data.**

|  | **Optimal care vs. suboptimal care**  **Low GRACE risk** | | **Optimal care vs. suboptimal care**  **Intermediate GRACE risk** | | **Optimal care vs. suboptimal care**  **High GRACE risk** | |
| --- | --- | --- | --- | --- | --- | --- |
|  | **aHR** | **Difference in AMR/100** | **aHR** | **Difference in AMR/100** | **aHR** | **Difference in AMR/100** |
| **HR over total follow-up time** | 0.81 (0.65-0.99) | -0.01 (-0.01 to 0.00) | 0.69 (0.61-0.79) | -0.02 (-0.03 to -0.02) | 0.69 (0.62-0.78) | -0.04 (-0.01 to -0.03) |
| **Time varying HR at set follow-up times** | | | | | | |
| 30 days | 0.95 (0.75-1.30) | -0.01 (-0.04 to 0.03) | 0.76 (0.65-0.92) | -0.10 (-0.16 to -0.04) | 0.93 (0.82-1.08) | -0.05 (-0.14 to 0.04) |
| 1 year | 0.78 (0.56-1.28) | -0.01 (-0.03 to 0.01) | 0.76 (0.61-1.01) | -0.04 (-0.08 to -0.01) | 0.67 (0.57-0.82) | -0.13 (-0.18 to -0.08) |
| 2 years | 0.77 (0.52-1.47) | -0.01 (-0.03 to 0.01) | 0.79 (0.61-1.11) | -0.03 (-0.06 to 0.00) | 0.68 (0.57-0.86) | -0.09 (-0.13 to -0.05) |
| 3 years | 0.77 (0.51-1.60) | -0.01 (-0.03 to 0.01) | 0.81 (0.62-1.16) | -0.02 (-0.05 to 0.01) | 0.70 (0.58-0.88) | -0.06 (-0.09 to -0.03) |
| 4 years | 0.77 (0.50-1.69) | -0.01 (-0.02 to 0.01) | 0.83 (0.63-1.20) | -0.02 (-0.04 to 0.01) | 0.71 (0.60-0.89) | -0.05 (-0.07 to -0.02) |
| 5 years | 0.77 (0.50-1.77) | -0.01 (-0.02 to 0.01) | 0.84 (0.64-1.22) | -0.01 (-0.03 to 0.01) | 0.73 (0.61-0.91) | -0.04 (-0.06 to -0.02) |
| 6 years | 0.78 (0.49-1.83) | -0.01 (-0.02 to 0.01) | 0.85 (0.65-1.23) | -0.01 (-0.03 to 0.01) | 0.74 (0.62-0.91) | -0.03 (-0.05 to -0.01) |
| 7 years | 0.78 (0.49-1.87) | -0.01 (-0.02 to 0.01) | 0.86 (0.66-1.25) | -0.01 (-0.03 to 0.01) | 0.75 (0.63-0.92) | -0.03 (-0.04 to -0.01) |
| 8 years | 0.78 (0.49-1.91) | -0.01 (-0.02 to 0.01) | 0.87 (0.67-1.25) | -0.01 (-0.02 to 0.01) | 0.75 (0.63-0.93) | -0.02 (-0.04 to -0.01) |
| ^*^Including referral for cardiac rehabilitation, smoking cessation advice and dietary advice. | | | | | | |

**References**

1. Van de Werf F, Ardissino D, Betriu A, Cokkinos DV, Falk E, Fox KA, Julian D, Lengyel M, Neumann F-J, Ruzyllo W. Management of acute myocardial infarction in patients presenting with ST-segment elevation. Eur Heart J 2003;**24**(1):28-66.

2. Bassand J-P, Hamm CW, Ardissino D, Boersma E, Budaj A, Fernández-Avilés F, Fox KA, Hasdai D, Ohman EM, Wallentin L. Guidelines for the diagnosis and treatment of non-ST-segment elevation acute coronary syndromes. Eur Heart J 2007;**28**(13):1598-1660.

3. Hamm CW, Bassand J-P, Agewall S, Bax J, Boersma E, Bueno H, Caso P, Dudek D, Gielen S, Huber K. ESC Guidelines for the management of acute coronary syndromes in patients presenting without persistent ST-segment elevation. Eur Heart J 2011;**32**(23):2999-3054.

4. Dondo TB, Hall M, Timmis AD, Gilthorpe MS, Alabas OA, Batin PD, Deanfield JE, Hemingway H, Gale CP. Excess mortality and guideline-indicated care following non-ST-elevation myocardial infarction. Eur Heart J Acute Cardiovasc Care 2016:2048872616647705.

5. Hall M, Dondo TB, Yan AT, Goodman SG, Bueno H, Chew DP, Brieger D, Timmis A, Batin PD, Deanfield JE. Association of clinical factors and therapeutic strategies with improvements in survival following non–ST-elevation myocardial infarction, 2003-2013. JAMA 2016;**316**(10):1073-1082.

6. Sterne JA, White IR, Carlin JB, Spratt M, Royston P, Kenward MG, Wood AM, Carpenter JR. Multiple imputation for missing data in epidemiological and clinical research: potential and pitfalls. BMJ 2009;**338**:b2393.
